# A Fluorescence-Polarization-Based Lipopolysaccharide–Caspase-4 Interaction Assay for the Development of Inhibitors

**DOI:** 10.3390/molecules27082458

**Published:** 2022-04-11

**Authors:** Jinsu An, So Yeon Kim, Eun Gyeong Yang, Hak Suk Chung

**Affiliations:** 1 Chemical and Biological Integrative Research Center, Biomedical Research Division, Korea Institute of Science and Technology, Seoul 02792, Korea; lhyun0506@gmail.com (J.A.); soyeonkim@kist.re.kr (S.Y.K.); eunyang@kist.re.kr (E.G.Y.); 2 Division of Bio-Medical Science & Technology, KIST School, Korea University of Science and Technology, Seoul 02792, Korea

**Keywords:** caspase-4, lipopolysaccharides, caspase activation and recruitment domain (CARD), non-canonical inflammasome, high-throughput screening, fluorescence polarization

## Abstract

Recognition of intracellular lipopolysaccharide (LPS) by Caspase-4 (Casp-4) is critical for host defense against Gram-negative pathogens. LPS binds to the N-terminal caspase activation and recruitment domain (CARD) of procaspase-4, leading to auto-proteolytic activation followed by pro-inflammatory cytokine release and pyroptotic cell death. Aberrant hyper-activation of Casp-4 leads to amplification of the inflammatory response linked to sepsis. While the active site of a caspase has been targeted with peptide inhibitors, inhibition of LPS–Casp-4 interaction is an emerging strategy for the development of selective inhibitors with a new mode of action for treating infectious diseases and sepsis induced by LPS. In this study, a high-throughput screening (HTS) system based on fluorescence polarization (FP) was devised to identify inhibitors of the LPS and Casp-4 interaction. Using HTS and IC_50_ determination and subsequently showing inhibited Casp-4 activity, we demonstrated that the LPS–Casp-4 interaction is a druggable target for Casp-4 inhibition and possibly a non-canonical inflammatory pathway.

## 1. Introduction

LPS is a glycolipid found in the outer membrane of nearly all Gram-negative bacteria [1]. This unique molecule is a well-known pathogen-associated molecular pattern that activates the host innate immune response and is recognized by Toll-like receptor 4/myeloid differentiation 2 (TLR4/MD2) [2]. Exposure of the host to an excessive amount of LPS can lead to uncontrolled inflammation and eventually sepsis [3], which is a leading cause of death in intensive care units. Therefore, molecules such as Eritoran, a lipid A mimetic antagonist, and TAK-242 (resatorvid) that inhibit the TLR4/MD2 signaling pathway have been developed to treat sepsis, but their clinical trials have failed [4,5].

While TLR4/MD2-dependent signaling induces transcription of pro-inflammatory cytokines and type I interferons in response to extracellular LPS [3,6], a type of inflammatory cell death termed pyroptosis accompanied by release of pro-inflammatory cytokines has been reported [7,8,9] in response to intracellular LPS released from outer membrane vesicles or intracellular Gram-negative bacteria. While Caspase-1 is activated via recruitment to the NLRP3 inflammasome, human Caspase-4 (Casp-4) and Caspase-5 (Casp-5) and the mouse orthologue Caspase-11 (Casp-11) are responsible for the recognition of intracellular LPS. Casp-4, -5, and -11 belong to a family of cysteine proteases and are classified as inflammatory caspases. These enzymes are synthesized as a latent zymogen consisting of the caspase activation and recruitment domain (CARD) and large and small subunits. Casp-4 and Casp-11 are recruited to intracellular LPS by guanylate-binding proteins [10,11] or galectin-3 [12], auto-processed, and activated via an interaction with LPS through their N-terminal CARD [9]. Activated Casp-4/-11 cleaves Gasdermin D, a pore-forming protein that executes pyroptotic cell death, and induces activation of the NLRP3 inflammasome followed by maturation of pro-interleukin (IL)-18 and pro-IL-1β [13,14,15].

Morbidity caused by LPS challenge is significantly delayed in Casp-11^−/−^ mice compared with both wild-type and Tlr4^−/−^ mice [7]. This suggests that Casp-11 plays pivotal roles in the morbidity of LPS-induced sepsis. Furthermore, Casp-11 plays important roles in many inflammation-related diseases, including inflammatory bowel diseases, multiple sclerosis, Parkinson’s disease, and rheumatoid arthritis [16]. Therefore, Casp-4 in humans has been suggested to be a promising target for the treatment of these diseases. The fact that several caspases share the same substrates, however, hampers successful development of inhibitors that specifically target the active site of Casp-4. In this regard, we focused on targeting the intrinsic activation of Casp-4 by LPS to develop Casp-4 inhibitors. In this study, we devised a high-throughput screening (HTS) system based on fluorescent polarization that monitors the inhibition of LPS and Casp-4 interaction by small molecules. Using the HTS system, we demonstrated that small molecules targeting LPS–Casp-4 interaction inhibit Casp-4 activation. The results suggest that searching for inhibitors of LPS–Casp-4 interaction might be an emerging strategy for inhibiting inflammation by LPS.

## 2. Results

### 2.1. CARD (1–80) of Caspase-4 Is a Necessary and Sufficient Domain for LPS Recognition

**Figure 1:**
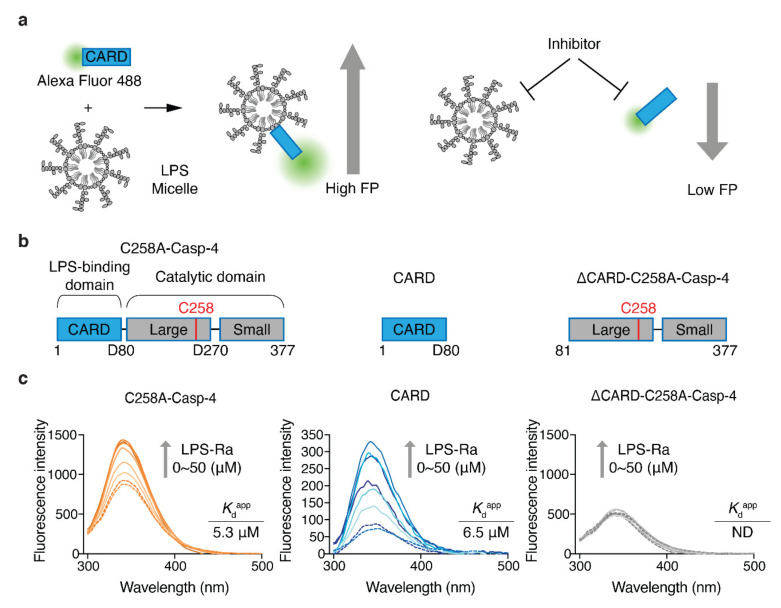
Design of an FP-based assay to monitor and screen inhibitors of the LPS–CARD interaction. (**a**) Schematic diagrams of an FP-based assay to monitor LPS/CARD binding and the effects of inhibitors. (**b**) Schematic domain structures of C258A-Casp-4, CARD, and ∆CARD-C258A-Casp-4. (**c**) Intrinsic Trp emission spectra of full-length C258A-Casp-4 (four Trp residues), CARD (one Trp residue), and ∆CARD-C258A-Casp-4 (three Trp residues) in the presence of varying concentrations of LPS-Ra (0–50 µM). The data are representative of three independent experiments.

To increase the efficiency of inhibitor discovery, we needed to develop an HTS system that monitors the LPS–Casp-4 interaction and its inhibitory compounds (Figure 1a). Previously, we reported that Casp-4 and its CARD (1–80 aa) bind to LPS micelles and form a complex with a high molecular weight [17]. This observation led us to devise an FP-based interaction assay. In contrast to the high molecular weight of Casp-4 (43.3 kDa), CARD is a relatively small protein (9.4 kDa) and is therefore suitable for the development of an FP-based assay. Although Casp-4 interacts with LPS through its CARD, it is unknown whether the CARD–LPS interaction represents all of the binding affinity of Casp-4/LPS. To investigate this, we constructed catalytically inactive full-length Casp-4 (1–377, C258A variant) (C258A-Casp-4), CARD (1–80), and Casp-4 lacking CARD (81–377, C258A) (ΔCARD-C258A-Casp-4) (Figure 1b) and compared their apparent dissociation constant (K_d_^app^) values by measuring tryptophan (Trp) fluorescence spectra (Figure 1c). The fluorescence emission spectrum of a Trp residue in an LPS-binding protein or antimicrobial peptide targeting the outer membrane of Gram-negative bacteria tends to exhibit a blue-shift and the fluorescence intensity also increases upon binding of LPS when a solvent-exposed Trp fluorophore is inserted into the hydrophobic environment of an LPS micelle. The K_d_^app^ can be derived from fluorescence emission spectra [18].

CARD has a Trp residue at position 37, which is predicted to be exposed to solvent in a model built by trRosetta [19] (Figure S1a from Supplementary Materials). Based on this prediction, we measured the fluorescence spectra and fluorescence intensities of C258A-Casp-4 (four Trp residues) and CARD (one Trp residue) in the presence of varying concentrations of LPS-Ra. Binding of LPS induced a blue-shift (2.05 and 11.8 nm for C258A-Casp-4 and CARD, respectively) and increased the fluorescence intensity in a dose-dependent manner (Figure 1c). The K_d_^app^ values calculated from the fluorescence spectra of C258A-Casp-4 and CARD were 5.3 ± 0.5 µM and 6.5 ± 0.8 µM, respectively (Figure 1c and Figure S1b from Supplementary Materials). In addition, surface plasmon resonance experiments with immobilized LPS O55:B5 ligand revealed that C258A-Casp-4 and CARD had similar dissociation constants of 2.4 and 2.1 µM, respectively (Figure S1c from Supplementary Materials). However, neither method detected the interaction of ΔCARD-C258A-Casp-4 (three Trp residues) and LPS (Figure 1c and Figure S1b,c, right panel from Supplementary Materials). Therefore, we concluded based on the quantitative data that LPS/CARD represents all the binding of LPS/Casp-4 and can be used as a ‘tracer’ of binding between LPS and Casp-4 in the FP-based assay (Figure 1a).

### 2.2. Development of the FP-Based Assay and Optimization of the Assay Conditions for HTS

**Figure 2:**
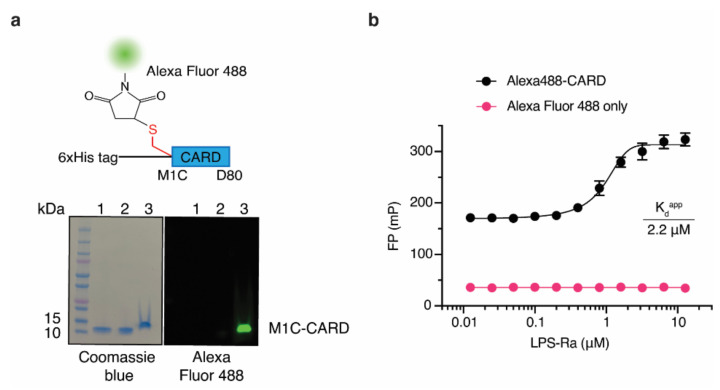
An FP-based assay using Alexa488-CARD and LPS-Ra. (**a**) Schematic structure of Alexa488-CARD and Coomassie blue staining (left) and fluorescence (right) images of an SDS-PAGE gel of M1C-CARD purified by Ni-NTA column chromatography (lane 1) and Superdex200 column chromatography (lane 2) and after labeling with Alexa Fluor 488 (lane 3). (**b**) FP binding curves of 50 nM Alexa488-CARD or dye only incubated with varying concentrations of LPS-Ra. Standard deviations presented in the graph were calculated from three independent experiments.

So far, native PAGE, SPR, and Gel filtration chromatography have been used to measure LPS and Casp-4 interactions. Even though SPR has higher sensitivity than the FP-based assay, these methods have limitations in their applicability to the HTS format. Moreover, as a ratiometric method, FP-based assays have advantages over other light-based assays for developing HTS assays that are relatively insensitive to instrumental interferences, including internal filter effects and absorption interferences [20]. Since site-specific labeling of fluorescent dye on the tracer reduces batch-to-batch variation and increases the reproducibility of the FP-based assay, we took advantage of the fact that CARD lacks cysteine. Therefore, we designed tracers in a site-specific manner by inserting a cysteine residue and labeled it with thiol-reactive dyes. To explore appropriate positions for insertion, three variants that contained a cysteine residue at the N-terminus (Cys-His-CARD), at the C-terminus (His-CARD-Cys), or between the N-terminal His_6_ tag and CARD (His-Cys-CARD, M1C-CARD) were generated and purified (Figure S2a from Supplementary Materials). With these CARD variants, we evaluated the site-specific labeling efficiency using the thiol-reactive fluorescent dye Alexa Fluor 488 (Figure S2b from Supplementary Materials). M1C-CARD had the highest labeling efficiency (86%) under the same labeling conditions (Figure S2 from Supplementary Materials); therefore, Alexa Fluor 488-labeled M1C-CARD (Alexa488-CARD) was selected as a ‘tracer’ (Figure 2a) to establish our FP-based assay. Since Alexa488-CARD has a molecular weight of 12.2 kDa, Alexa488-CARD exhibited a background FP signal in the absence of LPS-Ra. However, it showed a sigmoidal FP binding curve as the LPS-Ra concentration increased (Figure 2b). The K_d_^app^ determined from the FP-based assay for Alexa488-CARD and LPS-Ra was 2.2 ± 0.2 µM when 50 nM Alexa488-CARD was used (Figure 2b).

In order to efficiently screen inhibitors of the LPS–Casp-4 interaction, we needed to develop an HTS assay system that showed the maximum differences in the FP signal between CARD alone and LPS-bound CARD and robust readouts within a wide range (0–10%) of concentrations of DMSO, which is frequently used to dissolve chemical compounds. To establish conditions, we optimized additives and the pH, incubation duration, and DMSO concentration of the assay. Bovine serum albumin and NP40 additives were reported to improve the detection limit of the FP signal by reducing non-specific tracer absorption on the surfaces of wells and aggregation of the tracer itself [21]. However, in our assay system, these additives compromised LPS–CARD binding (Figure S3a from Supplementary Materials), probably due to disaggregation of LPS micelles induced by detergent or albumin [22,23]. In addition, the presence of divalent cations such as Mg^2+^ and Ca^2+^ interrupted LPS–CARD binding because electrostatic interactions are one of the major forces that mediate this binding [9] (Figure S3a from Supplementary Materials). We further optimized the concentration of the tracer (Figure S3b from Supplementary Materials), the pH of the buffer (Figure S3c from Supplementary Materials), and the incubation duration (Figure S3d from Supplementary Materials) to achieve the largest and most reliable dynamic range of FP values from Alexa488-CARD alone (low) to LPS micelles bound to Alexa488-CARD (high). Through the optimization experiments, we set the conditions to pH 7.0, 150 mM NaCl, and 37 °C with 50 nM Alexa488-CARD. Next, we evaluated the compatibility of DMSO. Although the dynamic range of FP values slightly decreased as the concentration of DMSO increased, the K_d_^app^ values remained within similar ranges up to 10% DMSO (Figure S3e from Supplementary Materials). Finally, 1.6 µM LPS-Ra for HTS was selected as the minimum concentration necessary to achieve a Z’ factor of ~0.7, a statistical cutoff value that provides reproducible separation of hits and controls [24], within a wide range of DMSO concentrations (Figure S3f from Supplementary Materials).

### 2.3. High-Throughput Screening Using the HTS System and IC_50_ Determination of Hit Compounds

**Figure 3:**
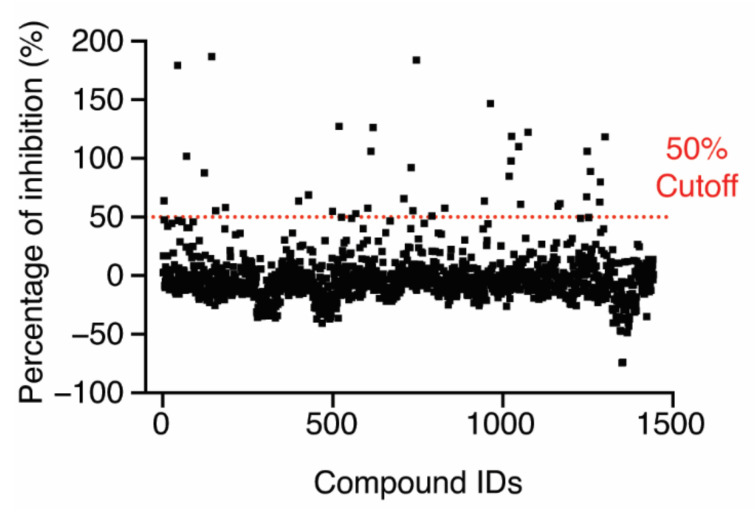
Screening of an FDA-approved drug library (1443 compounds) for inhibition of the LPS–CARD interaction. The dashed line indicates 50% inhibition in the presence of 10 µM compound.

After establishing the HTS conditions, we screened an FDA-approved drug library of 1443 compounds and selected initial hits that inhibited 50% of the FP signal (Figure 3). Among the initial hits, we excluded a pan-assay interference compound [25] and four fluorescence compounds that have similar excitation and emission spectra to Alexa488-CARD, after which 32 compounds remained (hit rate, ~2.2%) (Supplementary source data). The IC_50_ value of each hit was determined using the FP-based competition assay employing 6.4 µM LPS-Ra, which was a higher concentration than the K_d_^app^ value (4.1 ± 0.9 µM with 2% DMSO) (Figure S3 from Supplementary Materials), and 50 nM Alexa488-CARD with serially diluted hit compounds (0.022–50 µM). Nine of the thirty-two compounds induced a dose-dependent decrease in FP signals with micro-molar IC_50_ values (Figure S4 from Supplementary Materials).

### 2.4. Validation of Hit Compounds Using the Caspase-4 Activation Assay

**Figure 4:**
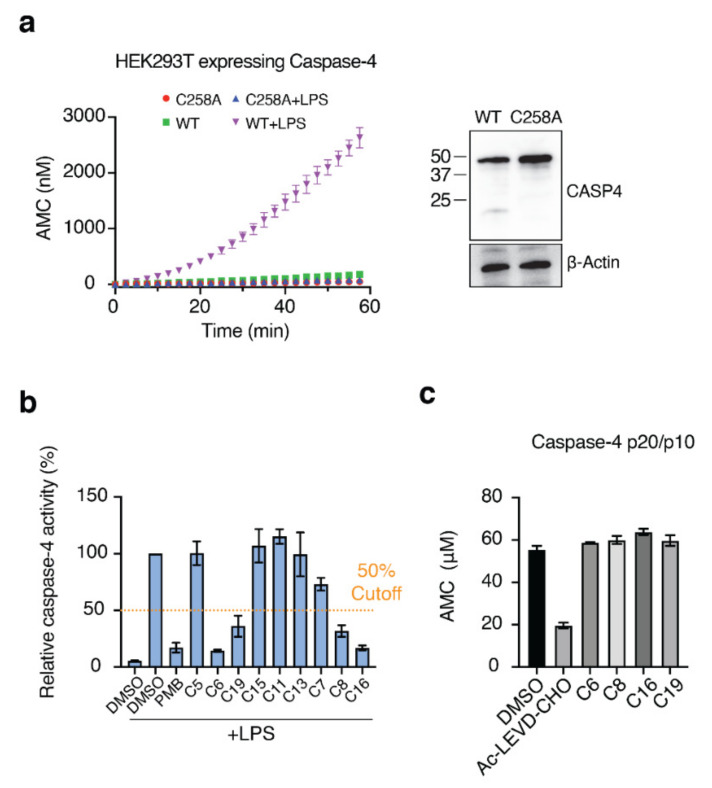
Four of the nine compounds inhibit the catalytic activity of Casp-4 by interrupting LPS–Casp-4 binding. (**a**) HEK293T cells were transiently transfected with p3xFLAG-CMV-10 Casp-4 or C258A-Casp-4. Cell lysates were assayed for Casp-4 activity using the substrate Ac-WEHD-AMC in the presence or absence of the activator LPS (left). Expression of recombinant proteins was analyzed by immunoblot (right). (**b**) Inhibition of Casp-4 activation by hit compounds was measured at 1 h. The dashed line indicates 50% inhibition. (**c**) Activity assay of activated Casp-4 (p20/p10) in the presence of the four compounds. Ac-LEVD-CHO was used as a competitive inhibitor of Ac-WEHD-AMC.

**Figure 5:**
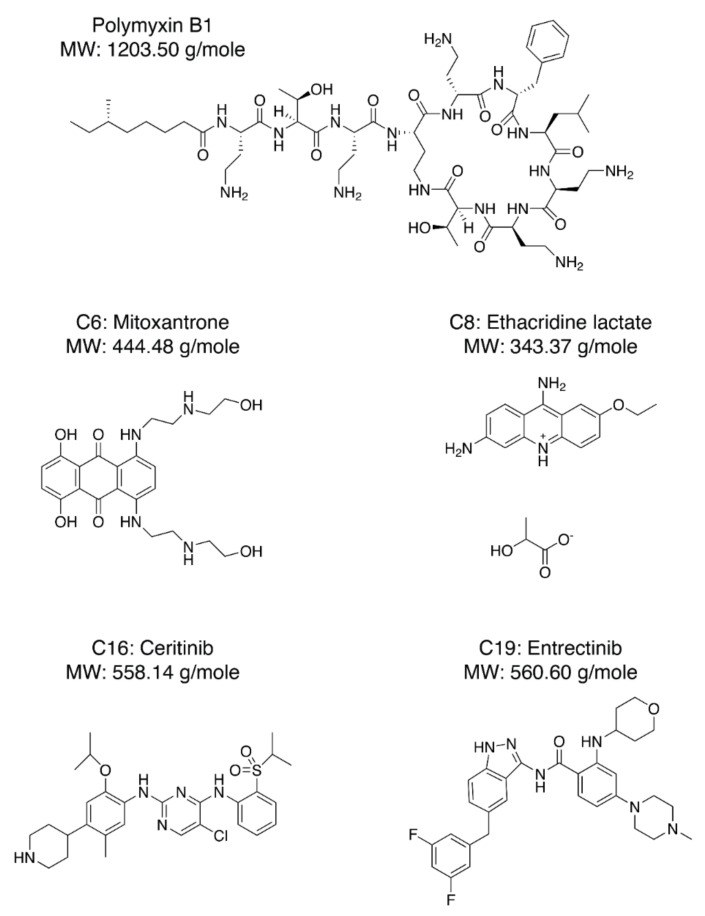
Chemical structures of PMB (Polymyxin B1), C-6 (Mitoxantrone), C-8 (Ethacridine lactate), C-16 (Ceritinib), and C-19 (Entrectinib).

Next, we needed to validate the abilities of the hit compounds to inhibit Casp-4 activation. If the hit compound inhibits the interaction between LPS and CARD, it will inhibit subsequent activation of Casp-4 and its catalytic activity. Accordingly, HEK293T cells were transfected with full-length wild-type Casp-4 or a catalytically inactive Casp-4 variant (C258A-Casp-4) and their activities were measured using the fluorogenic peptidyl substrate Ac-WEHD-AMC. To eliminate the discrepancy in the protein, we measured the expression levels of Casp-4 and C258A-Casp-4 by Western blotting and found the C258A-Casp-4 expression to be slightly higher than that of Casp-4 (Figure 4a). Hydrolysis of Ac-WEHD-AMC was not obvious when Casp-4 or C258A-Casp-4 was incubated in the absence of LPS; however, the activity of Casp-4, but not of C258A-Casp-4, was dramatically enhanced in the presence of LPS (Figure 4a). Using this validated assay, we tested the inhibition efficacies of the nine hit compounds in comparison with the negative control DMSO. As an inhibitor of LPS–Casp-4 interaction has been found, we wondered whether polymyxin B (PMB, Figure 5), an LPS-sequestering antibiotic, could inhibit LPS–Casp-4 interaction and Casp-4 activation induced by LPS. As shown in Figure 4b, PMB inhibited the Casp-4 activity induced by LPS. Therefore, we used PMB as a positive control in the activity assay. Four of the nine compounds inhibited Casp-4 activity by more than 50% at a concentration of 50 µM after 1 h (Figure 4b). Inhibition by these four compounds was also observed in a time-course of Casp-4 activity (Figure S5 from Supplementary Materials). In contrast, inhibition of Ac-WEHD-AMC hydrolysis by the compounds was not observed when activated Casp-4 (p20/p10) lacking CARD was used (Figure 4c). These data demonstrate that inhibition of the LPS–Casp-4 interaction is a new strategy for developing a specific drug that inhibits non-canonical inflammasome formation and sepsis induced by LPS. C6 (Mitoxantrone, Figure 5) is an antineoplastic agent that inhibits a type II topoisomerase via DNA intercalation for cancer treatment as well as expression of pro-inflammatory cytokines by LPS-stimulated astrocytes, and is approved for the treatment of multiple sclerosis [26]. C8 (ethacridine lactate, Figure 5) is primarily used as a disinfectant, but is also reported to shift the immune reaction toward a Th1-type response by modulating cytokine production, and activates macrophages to kill bacteria [27]. C16 (Ceritinib) and C19 (Entrectinib) are both antineoplastic agents for the treatment of non-small cell lung cancer (Figure 5). While the four selected compounds have known primary cellular targets at nanomolar concentrations, this study suggests that these compounds or their derivatives may have another pharmacological target during infection with Gram-negative bacteria.

## 3. Discussion

Caspases belong to a cysteine protease family and play important roles in apoptosis and inflammatory responses. Although inhibitors targeting the active sites of caspases have been developed, it remains challenging to develop subtype-specific inhibitors. In this study, we asked whether the LPS–Casp-4 interaction could be a targetable interaction by small molecules. In order to answer the question, we first developed an FP-based assay that monitors the LPS–Casp-4 interaction. Then, by using the FP-based HTS assay, calculating potencies of inhibitory hits, and performing an inhibition assay for Casp-4 activity, we found that 4 of the 1443 compounds as well as PMB inhibited Casp-4 activity by interrupting LPS–Casp-4 binding. Since these four compounds have a primary target in the cell in the nanomolar range, it would be challenging to use them as drugs targeting LPS–Casp-4 interaction in the micromolar range. However, these results demonstrate for the first time that the interaction between LPS and Casp-4 could be a drug discovery target for the development of Casp-4-selective inhibitors. In addition, our optimized HTS assay can be readily utilized to identify drug candidates for endotoxin-induced inflammatory disease and sepsis.

## 4. Materials and Methods

The Ac-WEHD-AMC was obtained from Enzo-life science (Farmingdale, NY, USA). The FDA-Approved drug library was obtained from Selleckchem (Houston, TX, USA). β-mercaptoethanol (2ME) and Tween20 were obtained from Bio-Rad (Hercules, CA, USA). The LPS-Ra mutant, LPS O55:B5, Digitonin, DMSO, 7-Amino-4-methylcoumarin (AMC), and all other chemicals were obtained from Sigma-Aldrich (Seoul, South Korea) unless stated otherwise.

### 4.1. Recombinant Protein Expression and Purification

Recombinant proteins were expressed and purified as described previously [17]. Full-length Caspase-4 C258A variant (C258A-Casp-4) and CARD (1–80) genes were cloned into pET28b vector with an N-terminal His6 tag using NdeI and HindIII restriction sites. CARD-truncated Caspase-4 (81–377, C258A) (ΔCARD-C258A-Casp-4), activated Caspase-4 (81–377) (p20/p10), and cysteine-inserted CARD (1–80) variants (Cys-His-CARD, His-Cys-CARD (M1C-CARD), and His-CARD-Cys) were constructed by QuikChange mutagenesis following the manufacturer’s protocol (Agilent Technologies, Santa Clara, USA). All constructs were expressed from ClearColi BL21(DE3) (Lucigen, Middleton, WI, USA) cells grown at 18 °C overnight in LB medium supplemented with 50 µg mL^−1^ kanamycin after induction with 0.2 mM isopropyl-β-d-thiogalactopyranoside when the optical density (OD600) reached 0.8. Cells were sonicated in buffer A (25 mM Tris-HCl pH 8.0, 300 mM NaCl, 20 mM imidazole) with 5 mM 2ME (1% Tween20 was supplemented for the full-length Casp-4 C258A). Thermostable CARD (1–80) and cysteine-inserted CARD variants were further treated at 70 °C for 30 min after sonication. The lysates were clarified by centrifugation at 12,000× *g* at 4 °C for 40 min. The supernatants were loaded on a HisTrap column (Cytiva) that was equilibrated with buffer A. Recombinant proteins were eluted by an increasing gradient of buffer B (25 mM Tris-HCl pH 8.0, 300 mM NaCl, 300 mM imidazole) after intensive washing (10 column volumes (CVs) of buffer A, 10 CVs of buffer A + 0.1% Tween 20, and 50 CVs of a buffer A and B mixture (9:1)). Proteins were further purified by a Superdex 200 gel-filtration column (Cytiva) with buffer C (50 mM Tris-HCl pH 7.0, 150 mM NaCl) at 4 °C.

### 4.2. Intrinsic Tryptophan Fluorescence Spectroscopy

Fluorescence spectra were obtained with a FluoroMate FS-2 fluorescence spectrophotometer (Sinco, Seoul, Korea) with a 5 mm path length quartz cuvette. One micromolar of recombinant proteins was incubated with increasing concentrations of LPS-Ra (ranging from 0 to 50 µM) in HBS-E buffer (10 mM HEPES pH 7.5, 150 mM NaCl, 3 mM EDTA) for 30 min at 37 °C. Samples were excited at 280 nm, and emission spectra from 300 nm to 500 nm were recorded with an excitation/emission slit width of 2.5/5 nm, respectively. The fluorescence intensity of samples was corrected by subtracting the LPS-only background intensities. Differences in fluorescence intensity at 334 nm were plotted as a function of LPS-Ra concentrations, and apparent dissociation constants (K_d_^app^) were obtained from a nonlinear regression curve fit as analyzed in GraphPad Prism 8.

### 4.3. Surface Plasmon Resonance (SPR)

SPR experiments were performed at 25 °C on a BIAcore T200 (Cytiva, Marlborough, MA, USA) at the Korean Basic Science Institute. A filtered and degassed running buffer (HBS-E) was prepared before use. An amine-derivative of LPS O55:B5 (NH-LPS) was prepared as described previously [28]. NH-LPS was immobilized on a CM5 sensor chip (Cytiva) using the amine coupling procedure and blocked with 1 M ethanolamine (pH 8.5). Indicated concentrations of purified proteins were injected, and the resulting sensorgrams were analyzed using BIAcore T200 Evaluation software.

### 4.4. Labeling of CARD Variants with Fluorescent Dye

Purified CARD variants with a cysteine residue were incubated in buffer C supplemented with a five-molar excess of Alexa-Fluor 488 C5 Maleimide (Invitrogen, Carlsbad, CA, USA) and a ten-fold excess of TCEP at 4 °C overnight. Un-labeled free dyes were excluded by a PD-10 desalting column (Cytiva) and a 10K centrifugal filter (Millipore, Billerica, MA, USA) until no dye was detected in the flow-through. According to the Lambert–Beer law, the labeling efficiency was calculated with absorbance values at 280 nm and 489 nm measured by a UV/Vis spectrophotometer (Biochrom Libra S22, Cambridge, UK). A correction factor of 0.11 was used to adjust the absorbance at 280 nm contributed by Alexa-Fluor 488.

### 4.5. Fluorescence Polarization Assays

Fluorescence polarization (FP) was measured by an Appliskan microplate reader (ThermoFisher, Waltham, MA, USA) with 485 ± 5 nm excitation and 535 ± 10 nm emission filters. The FP value was calculated according to the following equation: FP (mP) = 1000 × (I_s_ − G × I_p_)/(I_s_ + G × I_p_), where I_s_ is the parallel (same) emission intensity, I_p_ is the perpendicular emission intensity, and G is the grating factor of 0.877, which was experimentally determined, or 1 for HTS experiments. FP binding assays were performed using a black 96-well plate (SPL life science, Pocheon, Korea) with 200 µL of the reaction mixture per well. Reaction mixtures containing 50 nM of Alexa 488-CARD (tracer) with increasing concentrations of LPS-Ra (0–12.8 µM; the molecular weight of LPS-Ra was estimated to be 3835 g/mole) in buffer C or indicated buffers were incubated at 37 °C for 30 min to 4 h before measurements. The FP values were plotted as a function of LPS-Ra concentrations, and apparent dissociation constants (K_d_^app^) were obtained from a nonlinear regression curve fit as analyzed in GraphPad Prism 8. FP competition assays were performed by mixing 50 nM of tracer, 6.4 µM of LPS-Ra, and 3-fold serially diluted hit compounds (50 to 0.022862 µM) in buffer C. Mixtures were incubated at 37 °C for 1 h before measurements. The FP values were plotted as a function of the compounds’ concentrations, and IC_50_ values were calculated by a sigmoidal curve fit as analyzed in GraphPad Prism 8. The Z’ factor was used to evaluate the reproducible separation of FP values of LPS and Alexa488-CARD binding and the control under varying concentrations (0 to 10%) of DMSO. The Z’ factor was calculated according to the following equation: 1 − (3SD_s_ + 3SD_p_)/|µ_s_ − µ_p_|, where SD_s_ and SD_p_ are the standard deviations, and µ_s_ and µ_p_ are the means of the FP values obtained from the sample and the positive (without LPS) control, respectively.

### 4.6. High-Throughput Screening

A total of 0.8 µL of the FDA-approved drug compound (2.5 mM dissolved in DMSO (1337 compounds) or water (106 compounds)) was transferred into each well of 96-well plates containing 50 nM of tracer and 1.6 µM of LPS-Ra by a pipetting machine (Mosquito, SPT Labtech) to achieve a final volume of 200 µL per well. Each plate had the negative (DMSO or water with LPS) and positive (DMSO or water without LPS) controls. FP was measured after 1 h of incubation at 37 °C using the Appliskan microplate reader. The final percentage of inhibition was calculated using the following equation: Inhibition (%) = 100 × ((mP_negative_ − mP_test compound_)/(mP_negative_ − mP_positive_)), where mP_negative_ is the FP value of the negative control, mP_postive_ is the FP value of the positive control, and mP_test compound_ denotes the FP values of samples containing compounds, respectively. We arbitrarily chose 50% inhibition as a threshold.

### 4.7. Casp-4 Activation Assay

Wild-type Casp-4 and C258A inactive variant genes were cloned into p3xFLAG-CMV-10 vector using KpnI and XbaI restriction sites. HEK293T cells cultured in DMEM medium (Welgene, Gyeongsan, Korea) supplemented with 10% heat-inactivated FBS (Gibco, Waltham, MA, USA), 100 U mL^−1^ penicillin, and 100 U mL^−1^ streptomycin were transfected with each construct using lipofectamine2000 (Thermo Fisher Scientific). After 24 h, cells were re-seeded in black, flat-bottomed 96-well culture plates (SPL life sciences) at 10^5^ cells per well. The culture supernatant was replaced after 2 hours of incubation for cell attachment with activity assay buffer (buffer C supplemented with 0.01% (*w/v*) digitonin, 10 mM DTT, 100 µM Ac-WEHD-AMC, 10 µM LPS-Ra, and 50 µM hit compounds or the same volume of DMSO). For active Casp-4 (p20/p10), 1 µM of purified p20/p10 was assayed with the same assay buffer. Hydrolysis of fluorogenic AMC was monitored using the Appliskan microplate reader at room temperature with 326 nm excitation and 460 nm emission filters at set time intervals. The amount of hydrolyzed free AMC was calculated with standard curves derived from serially diluted AMC in assay buffers containing each hit compound.

### 4.8. Immunoblot

Cell extracts were prepared using RIPA buffer (50 mM Tris-HCl pH 7.5, 150 mM NaCl, 1% Triton X-100, 1% Sodium deoxycholate, 0.1% SDS, 2 mM EDTA) supplemented with a protease inhibitor cocktail (Sigma). Proteins were subjected to SDS-PAGE and transferred to a PVDF membrane. Human Caspase-4 (#4450, Cell Signaling, Danvers, MA, USA) or beta actin (#MA5-15739, Invitrogen) antibodies were incubated with the PVDF membrane overnight at 4 °C. Blots were developed with HRP-conjugated secondary antibodies and visualized using enhanced chemiluminescence (ATTO KOREA, Daejeon, Korea).

## 5. Patents

H.S.C., J.A., S.Y.K., and E.G.Y. have filed patents for the drug screening method targeting LPS–Casp-4 interactions.

## Data Availability

Not applicable.

## Supplementary Materials

The following supporting information can be downloaded at: https://www.mdpi.com/article/10.3390/molecules27082458/s1, Figure S1: LPS–Caspase-4 (CARD) binding data using tryptophan fluorescence emission spectra or SPR sensorgrams; Figure S2: Optimization of the fluorescence dye labeling position in CARD; Figure S3: Optimization of the FP assay to apply for high-throughput inhibitor screening; Figure S4: IC50 of hit compounds determined with the FP assay; Figure S5: Inhibition of LPS-induced Casp-4 activation by four hit compounds in a time-course.

## Sample Availability

Samples of the compounds are available from the authors.
