# Supplementary material for: A Fluorescence-Polarization-Based Lipopolysaccharide–Caspase-4 Interaction Assay for the Development of Inhibitors
Source: Molecules. 2022 Apr 11;27(8):2458. doi: 10.3390/molecules27082458 (PMC9032125; doi:10.3390/molecules27082458)
Supplement: Supplementary file 1 [file molecules-27-02458-s001.zip › molecules-1656948-supplementary/molecules-1656948_Supplementary.pdf]

molecules

Supplementary Materials

A Fluorescence-Polarization-Based Lipopolysaccharide–Caspase-4 Interaction Assay for the Development of Inhibitors

Jinsu An [1,2], So Yeon Kim [1,2], Eun Gyeong Yang [1] and Hak Suk Chung [1,2,*]

[1] Chemical and Biological Integrative Research Center, Biomedical Research Division, Korea Institute of Science and Technology, Seoul 02792, Korea; lhyun0506@gmail.com (J.A.); soyeonkim@kist.re.kr (S.Y.K.); eunyang@kist.re.kr (E.G.Y.)
[2] Division of Bio-Medical Science & Technology, KIST School, Korea University of Science and Technology, Seoul 02792, Korea
* Correspondence: hschung@kist.re.kr

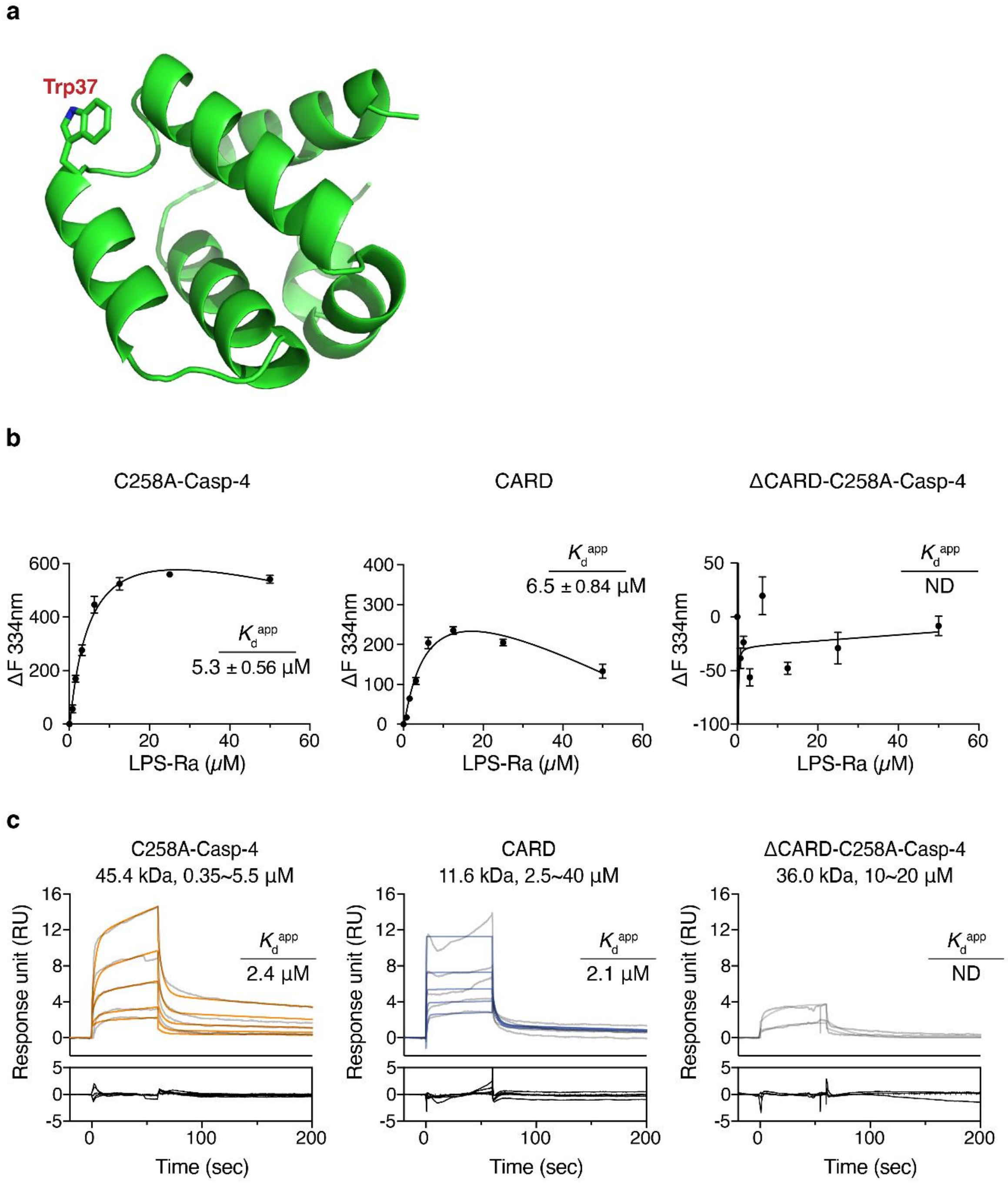

Figure S1. (a) A molecular model of CARD predicted by trRosetta[1]. (b) Saturation binding curves of C258A-Casp-4, CARD, and ΔCARD-C258A-Casp-4 with varying concentrations of LPS-Ra (0 - 50 μM) were derived from Trp fluorescence emission spectra shown in Fig. 1. Three independent experiments were presented as the mean ± standard deviation. (c) SPR sensorgrams of C258A-Casp-4, CARD, and ΔCARD-C258A-Casp-4 binding to *E. coli* LPS (O55:B5) immobilized on a CM5 sensor chip. Sensorgrams were obtained by varying concentrations of recombinant proteins indicated in Fig. S1c. Molecular weights of His6-tagged C258A-Casp-4, CARD, and CARD-C258A-Casp-4 are 45.4, 11.6, and 36.0 kDa, respectively. Residual plots were lie within reasonable limits around zero (except spikes at the beginning and end of the injection) indicating that the fitted curves are close to the experimental data.

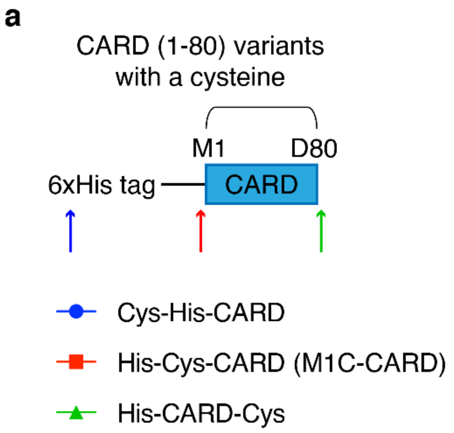

b

CARD (1-80) variants	Labeling efficiency (%)
Cys-His-CARD	24
His-Cys-CARD (M1C-CARD)	86
His-CARD-Cys	7.8

Figure S2. Optimization of labeling position in CARD. Schematic diagrams of CARD variants with a cysteine residue (a) and labeling efficiency of thiol-reactive dye (b).

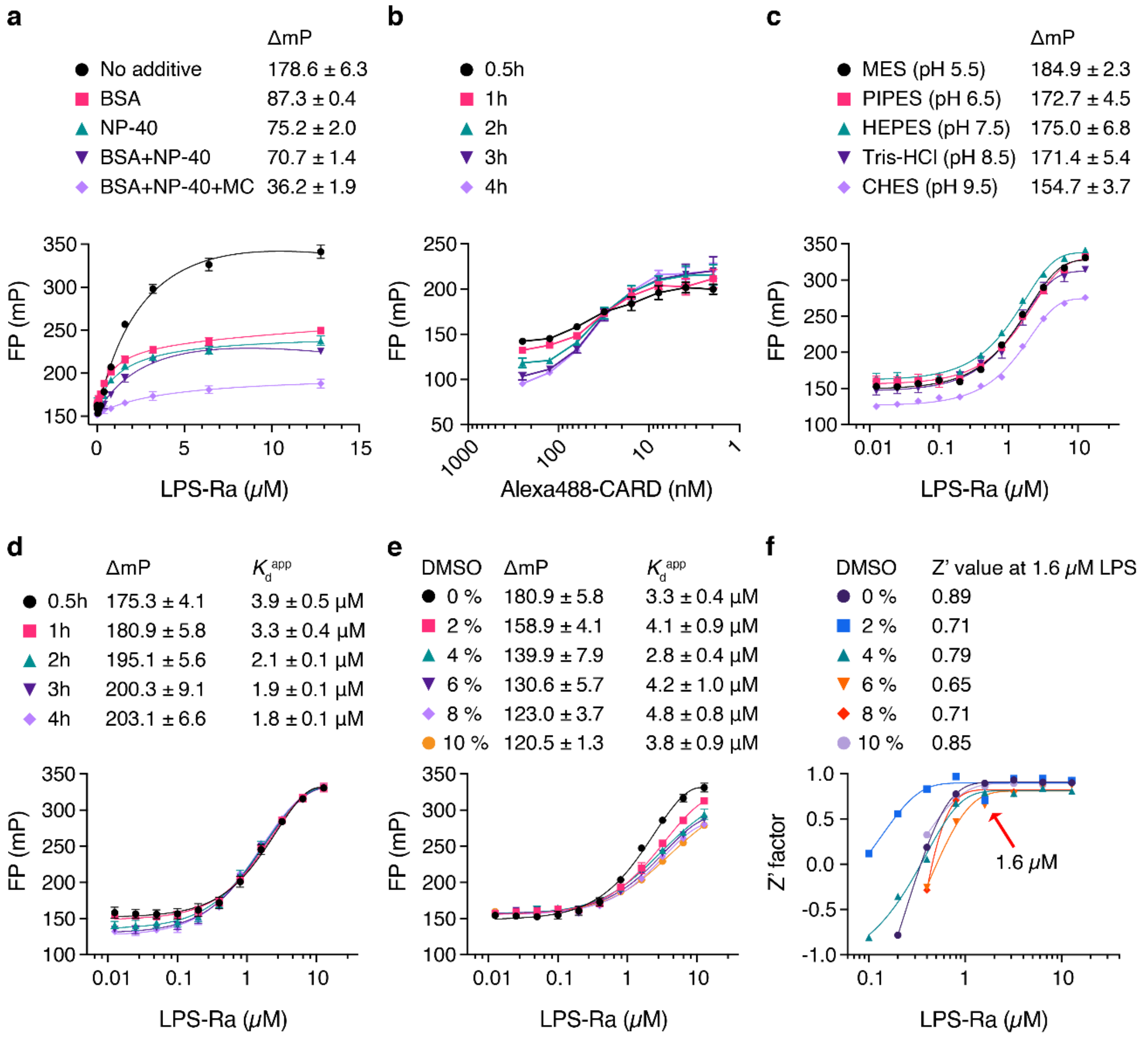

Figure S3. Optimization of FP assay to apply for high-throughput inhibitor screening. (a) FP binding curves in buffer C (50 mM Tris-HCl pH 7.0, 150 mM NaCl) supplemented with different additives; 0.1 mg/mL BSA, 0.01% NP-40, or 1 mM MC ($MgCl_2$ + $CaCl_2$). (b) The FP values of serially diluted Alexa488-CARD in buffer C were measured at 0.5 to 4 hours' incubation. (c) FP binding curves in five different pH buffers. (d) FP binding curves for 0.5 to 4 hours' incubation with varying concentrations of LPS-Ra in buffer C. (e) FP binding curves in buffer C supplemented with varying concentrations of DMSO. (f) Z' factors depending on LPS-Ra concentrations were evaluated in buffer C supplemented with 0 to 10% DMSO. The reactions were incubated at 37 °C for 1 hour unless stated. Three independent experiments are presented as the mean ± standard deviation in graphs (a-e).

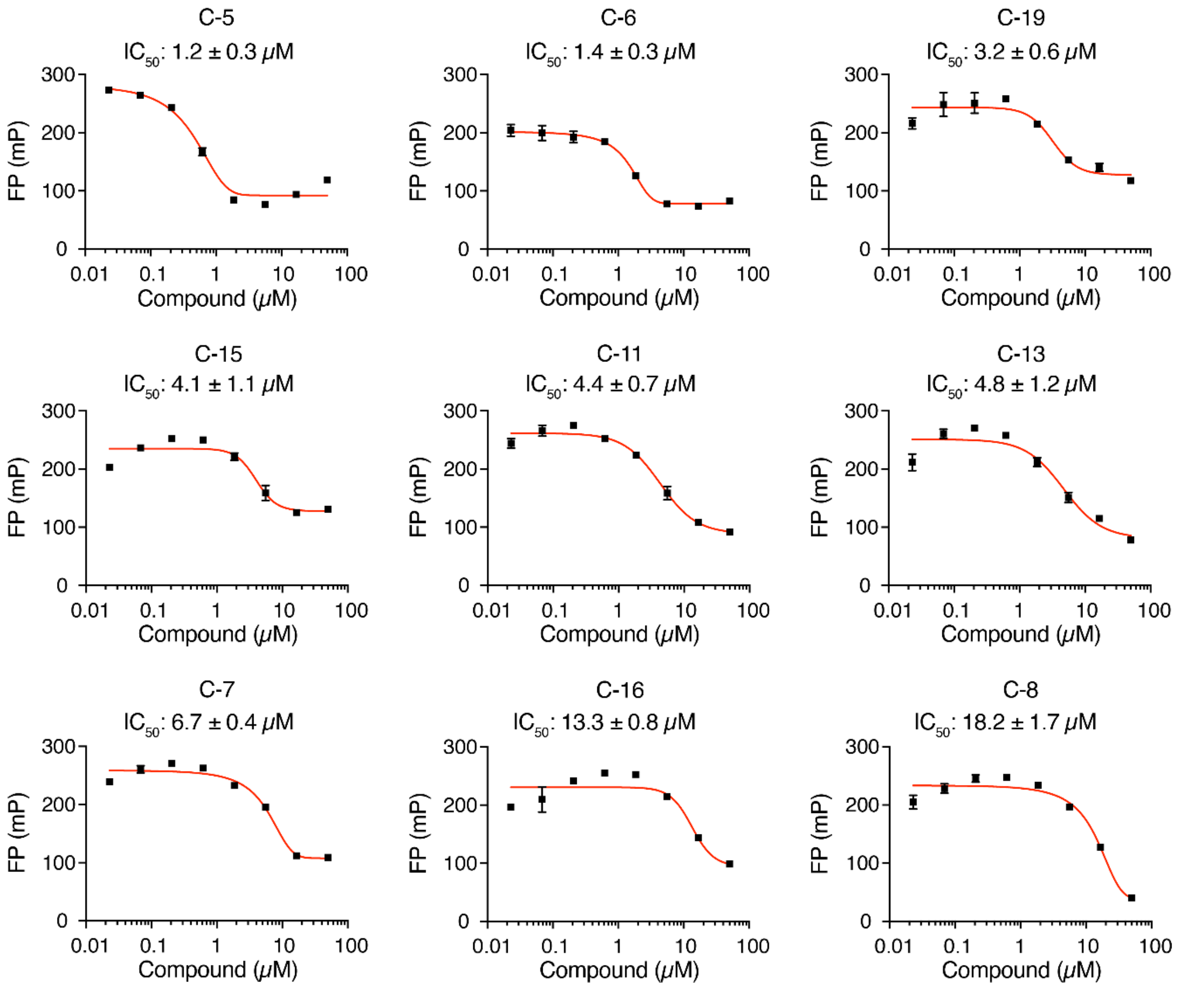

Figure S4. FP competition assays of hit compounds. A dose-response curve of C-5 (Crystal violet), C-6 (Mitoxantrone), C-19 (Entrectinib), C-15 (Sotrastaurin), C-11 (Eltrombopag Olamine), C-13 (Enzastaurin), C-7 (Eltrombopag), C-16 (Ceritinib), or C-8 (Ethacridine lactate) against LPS-Ra (6.4 μM) and Alexa488-CARD (50 nM) binding. IC_{50} values were determined from three independent experiments. Data are presented as the mean ± standard deviation.

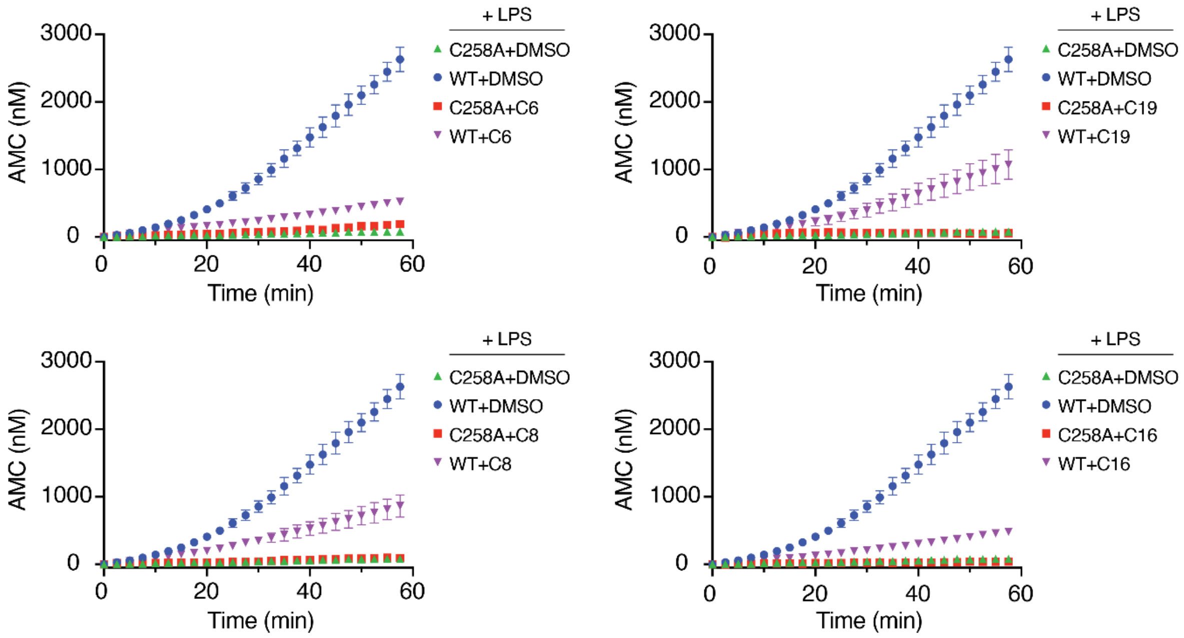

Figure S5. Inhibition of LPS-induced Casp-4 activation by four hit compounds in time course, C-6 (Mitoxantrone), C-8 (Ethacridine lactate), C-16 (Ceritinib), and C-19 (Entrectinib) were monitored by the AMC release from Ac-WEHD-AMC.

References:

1 Yang, J. Y. *et al.* Improved protein structure prediction using predicted interresidue orientations. *Proceedings of the National Academy of Sciences of the United States of America* **117**, 1496-1503, doi:10.1073/pnas.1914677117 (2020).
